# Post‐Treatment Strategies Toward High‐Quality Sb_2_Se_3_ Thin Films in Photovoltaic Applications

**DOI:** 10.1002/advs.202511387

**Published:** 2025-08-07

**Authors:** Qi Zhao, Rongfeng Tang, Shangfeng Yang, Tao Chen

**Affiliations:** ^1^ Hefei National Research Center for Physical Sciences at the Microscale, School of Chemistry and Materials Science University of Science and Technology of China Hefei Anhui 230026 P. R. China; ^2^ Institute of Deep Space Sciences Deep Space Exploration Laboratory Hefei 230088 China

**Keywords:** post‐treatment, Sb_2_Se_3_, solar cells

## Abstract

Antimony selenide (Sb_2_Se_3_) has attracted increasing attention as a promising photovoltaic absorber due to its superior optoelectronic properties and ample application potential in thin‐film solar cells. High‐performance Sb_2_Se_3_ solar cell is closely tied to the quality of Sb_2_Se_3_ active layer, which requires careful design of the interfacial and bulk defects, as well as crystallinity of the thin films. Postprocessing procedures show great potential to address defect issues and improve the conductivity of solar cells. Therefore, developing efficient and reliable post‐treatment techniques is crucial for advancing Sb_2_Se_3_ solar cell technology. In this review, recent post‐treatment methodologies are summarized toward high‐quality Sb_2_Se_3_ thin films, categorizing the strategies into two main types: thermal annealing (TA)‐related techniques and TA‐free techniques. Furthermore, the effects of these strategies are discussed on Sb_2_Se_3_ crystal characteristics, including defects, optoelectronic properties, and film morphology, all of which are closely related to device performance. Finally, the critical challenges and perspectives are proposed regarding this new solar cell materials, practical guidelines are also provided for fabricating high‐quality Sb_2_Se_3_ layers for highly efficient Sb_2_Se_3_ solar cells.

## Graphical abstract

This review provides a systematic summary of recent advancements in post‐treatment techniques for Sb_2_Se_3_ films and their applications in enhancing photovoltaic device efficiency. It covers relevant progress, identifies current challenges, and provides useful perspectives for future research directions.

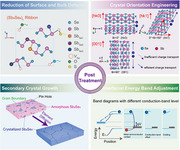

## 1 Introduction

Post‐treatment techniques play vital roles in the development of solar cells due to their ability to modulate morphology of active layer, passivate deep‐level defects, and improve interface quality. Particularly, post‐treatment processes are critical in achieving state‐of‐the‐art performance of thin‐film solar cells, such as perovskite solar cells (PSCs), copper indium gallium selenide (CIGS), and cadmium telluride (CdTe) solar cells. In PSCs, methods such as solvent annealing,^[^ ^1^, ^2^ ^]^ interface engineering,^[^ ^3^, ^4^ ^]^ and additive incorporation^[^ ^5^, ^6^ ^]^ are widely adopted to improve film quality and device stability. Benefiting from alkali metal doping (Na,^[^ ^7^ ^]^ K,^[^ ^8^ ^]^ Rb,^[^ ^9^ ^]^ Cs^[^ ^10^ ^]^), the local electrostatic environment of CIGS solar cells can be adjusted to promote grain growth and reduce recombination. In addition, sulfurization^[^ ^11^, ^12^ ^]^ and selenization processes^[^ ^13^ ^]^ are able to fine‐tune the absorber's bandgap and improve the carrier transport performance, which jointly contribute to highly efficient CIGS solar cells. In CdTe devices, the widely used CdCl_2_ postdeposition strategy^[^ ^14^ ^]^ is beneficial for facilitating Cl diffusion to activate grain boundary passivation and promote grain coalescence, ultimately improving carrier mobility.

In this broader context, post‐treatment strategies are equally indispensable for the development of emerging antimony selenide (Sb_2_Se_3_) solar cells. Over the past decade, Sb_2_Se_3_ has attracted increasing attention as a promising light‐harvesting material due to its superior optoelectronic properties and environmentally friendly characteristics.^[^ ^15^, ^16^ ^]^ Structurally, Sb_2_Se_3_ is a quasi1D material^[^ ^17^ ^]^ which has a high absorption coefficient exceeding 10⁵ cm^−1^ in the visible region.^[^ ^18^ ^]^ This structural uniqueness lies in the fact that the (Sb_4_Se_6_)_*n*_ ribbon terminated by (hk0) plane does not cause bond breakage, which suppresses the formation of detrimental dangling bonds and thus avoids nonradiative recombination.^[^ ^19^ ^]^ Based on these characteristics, the theoretical maximum efficiency of single‐junction Sb_2_Se_3_ solar cells is ≈32% according to the Shockley–Queisser limit theory.^[^ ^20^ ^]^ Since 2013, the PCE of Sb_2_Se_3_ thin‐film solar cells has steadily increased and now reaches 10.57%. Typically, the high‐efficiency Sb_2_Se_3_ solar cells consist of an Sb_2_Se_3_ absorber layer sandwiched between an electron‐transporting layer (ETL) and a hole‐transporting layer (HTL), with electrodes on both sides. Depending on the device architecture, we can assemble Sb_2_Se_3_ solar cells on transparent conductive oxide (TCO)‐coated glass substrates (superstrate configuration) or molybdenum‐coated glass substrates (substrate configuration). The performance of these devices strongly depends on the quality of photoactive films and their affinity and energy level alignment with the functional layers.

The Sb_2_Se_3_ photoactive layer plays a critical role in Sb_2_Se_3_ heterojunction thin film solar cells, performing functions, such as light absorption, photoinduced charge generation, and carrier separation. It should be emphasized that, the 1D crystal structure of Sb_2_Se_3_ demands vertical growth of (Sb_4_Se_6_)_*n*_ ribbons with dominant (hk1) planes to ensure efficient charge transport.^[^ ^21^ ^]^ However, the rapid crystal growth process often leads to defects at grain boundaries and within grains, which can cause severe nonradiative recombination during charge transport. Therefore, controlling the morphology and microstructure of Sb_2_Se_3_ thin films is key to reducing recombination losses and achieving high PCE. Recent studies have demonstrated that carefully designed postprocessing protocols‐ranging from chemical treatments^[^ ^22^, ^23^ ^]^ to thermal annealing^[^ ^24^, ^25^ ^]^ are crucial for fabricating uniform, pinhole‐free Sb_2_Se_3_ absorber layers with optimal microstructure. These treatments help mitigate defect formation, enhance crystallinity, and facilitate preferred crystal orientation, thereby significantly boosting power conversion efficiency (PCE). Indeed, some of the highest‐performing Sb_2_Se_3_ photovoltaic devices reported to date owe their success to meticulous post‐treatment steps, highlighting the central role of these techniques in device fabrication. However, it should also be noted that while such postprocessing can effectively improve crystallinity and crystal orientation, excessive grain growth or recrystallization may introduce film stress, cracks, or adhesion issues. Therefore, careful optimization of treatment conditions is essential to balance improved film quality with structural integrity and device stability. Therefore, a comprehensive review of post‐treatment techniques is essential for the research and subsequent development of Sb_2_Se_3_ solar cell community.

This review focuses on the effective post‐treatment techniques for improving Sb_2_Se_3_ solar cell performance. We first outlined the early technical developments in fabricating Sb_2_Se_3_ films and summarized the evolution of post‐treatment methods. Then, we discuss the mechanisms of various postprocessing techniques in improving film quality (including crystallinity, defects, morphology, etc.) and device performance. Finally, a forward‐looking perspective is provided on the future trends and challenges of optimizing Sb_2_Se_3_ absorber layers for next‐generation highly efficient photovoltaic devices.

## 2 High‐Quality Sb2Se3 Thin Films for High‐Performance Sb2Se3 Solar Cells

Basically, the grain size, orientation, crystallinity, and defect properties of Sb_2_Se_3_ thin films are key factors in device performance optimization. For polycrystalline thin films, their morphology depends on various factors, such as deposition parameters, substrate properties, and annealing conditions, which exhibit widely varying morphologies. Through precisely modulating Sb_2_Se_3_ crystallization kinetics via optimizing film processing procedures, high‐quality Sb_2_Se_3_ films with smooth and full coverage, high crystallinity, large grain size, and pinhole‐free can be obtained. Such thin films are expected to achieve low electrical shunt and suppress charge recombination, resulting in enhanced photovoltaic efficiency.

### 2.1 Development of Sb2Se3 Thin Film Fabrication Methods

**Figure 1:**
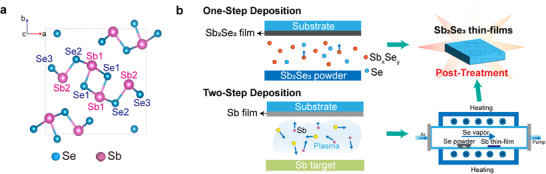
a) Crystal structure of Sb_2_Se_3_. b) Two types of fabrication methods for Sb_2_Se_3_ films: one‐step deposition method and two‐step sequential deposition method.

The anisotropy of Sb_2_Se_3_ crystal structure (**Figure** **1**a) increases the requirement for controllability of crystal orientation, as well as regulation of grain size and trap states, thereby posing a challenge to improving the efficiency of Sb_2_Se_3_ solar cells. Sb_2_Se_3_ thin‐film solar cells feature two kinds of configurations: substrate and superstrate architectures. In the substrate configuration, film layers are deposited in sequence starting from the substrate/Sb_2_Se_3_ back contact, followed by the deposition of an electron transport layer (ETL) and a top transparent electrode (TE). In this device, light is incident from the highly transparent TE layer.^[^ ^26^, ^27^ ^]^ This configuration allows the use of opaque substrates, such as commercially available rigid and flexible thin metallic foils, ceramics, and polymers. In the superstrate configuration, ETL/Sb_2_Se_3_ layer/hole transport layer (HTL) are deposited sequentially from the transparent electrode substrate side, with the metal electrode being deposited on top. In contrast to devices with substrate structures, the light is incident from the substrate side of superstrate devices to ensure sufficient absorption by the active layer.^[^ ^28^, ^29^ ^]^ This configuration requires the use of highly transparent substrates, as light must pass through the substrate to reach the absorber layer. The variations in deposition substrates for Sb_2_Se_3_ across different structures impose distinct requirements on the deposition techniques employed. In general, the fabrication methods for Sb_2_Se_3_ films can be categorized into two types: the one‐step deposition and the two‐step deposition methods (Figure 1b). With the innovation of processing techniques, the Sb_2_Se_3_ thin film quality, including crystal structure, film morphology, and surface coverage, have achieved dramatic improvements.

The one‐step deposition process is the most popular procedure for fabricating high‐quality Sb_2_Se_3_ films. This method offers a simplified approach to synthesis, minimizing complexity and potential contamination. In the early stages of Sb_2_Se_3_ photovoltaic research, solution processing techniques, especially spin coating method,^[^ ^30^, ^31^, ^32^ ^]^ plays a vital role. However, the efficiency of solution‐processed Sb_2_Se_3_ solar cells remains relatively low, primarily due to contamination risks from solvent impurities and difficulties in achieving compact and highly crystalline films. This situation leads to a shift toward more reliable physical vapor deposition methods that enhance control over film characteristics. Under this scenario, the physical vapor deposition methods have become more popular in Sb_2_Se_3_ solar cells. The one‐step physical preparation method for Sb_2_Se_3_ thin films typically involves the evaporation of Sb_2_Se_3_ powder, where both antimony (Sb) and selenium (Se) are simultaneously deposited onto a substrate in a controlled vacuum environment.^[^ ^27^, ^33^, ^34^, ^35^ ^]^ The simultaneous deposition of Se and Sb elements substance simplifies the fabrication process. This method allows for the direct formation of the desired phase with good uniformity and control over film thickness. However, the difference in melting points of the elements poses difficulties for this method in controlling stoichiometry and the formation of undesired phases. In contrast, the two‐step physical preparation method involves an initial deposition of Sb thin films, followed by the selenization process.^[^ ^36^, ^37^, ^38^ ^]^ In the second step, the Sb films are exposed to selenium vapor or a selenium‐containing atmosphere at elevated temperatures, allowing the formation of Sb_2_Se_3_ through diffusion and reaction. The sequential nature allows for better tuning of the composition and provides greater control over the film's stoichiometry. Moreover, the selenization process often leads to better crystalline quality, which is crucial for enhancing the material's optoelectronic properties and overall performance in solar cells. Nonetheless, the two‐step process is more time‐consuming and may even require additional equipment.

### 2.2 Evolution of Post‐Treatment Techniques Toward Sb2Se3 Layers

In both one‐step and two‐step sequential deposition methods, a variety of post‐treatment techniques are extensively employed to enhance the crystallinity, increase grain size, and modulate grain orientation of Sb_2_Se_3_ films. The grain growth and microstructural evolution of the films are intricately linked to the special post‐treatment conditions employed. Among these techniques, thermal annealing (TA) stands out as the most prevalent and widely utilized method in laboratory settings for device fabrication, playing a crucial role in the development of high‐quality Sb_2_Se_3_ films.^[^ ^39^, ^40^ ^]^ For traditional TA process, heating temperature and duration are the key parameters influencing the evolution of Sb_2_Se_3_ phase.^[^ ^41^, ^42^, ^43^ ^]^ However, straightforward TA post‐treatment of Sb_2_Se_3_ may lead to the formation of extensive defects at grain boundaries and interfaces, ultimately resulting in diminished crystal quality and misalignment of interfacial energy band. Integrating interface passivation strategy with traditional TA treatment is a recognized solution to improve device efficiency and stability.^[^ ^44^, ^45^ ^]^ Moreover, it is critical to note that Se element is prone to volatilization during the heating process, which easily cause Se vacancy (*V*_Se_) defects. The high‐density *V*_Se_ defects severely shorten the carrier lifetime of Sb_2_Se_3_ and lead to a large open‐circuit voltage (*V*_OC_) deficit in the device.^[^ ^46^, ^47^ ^]^ Given the significant impact of annealing atmosphere on crystal growth, the dominant effect of annealing conditions on device performance has also been extensively corroborated by experimental studies.^[^ ^48^, ^49^, ^50^ ^]^ Specifically, the S or Se atmosphere generated by sulfurization or selenization treatments can improve the conversion efficiency of Sb_2_Se_3_ solar cell by effectively reducing the concentration of *V*_Se_ defects.^[^ ^35^, ^51^ ^]^ Moreover, treating the film with specific solvents is beneficial for dissolving undesirable phases or increasing surface uniformity. Besides, various surface modifications can be achieved by exposing the material to a plasma environment, such as structural reconstruction, defect control, surface etching, etc., thereby improving wettability and conductivity.^[^ ^52^, ^53^ ^]^ Interface passivation treatment is a facile and widely used post‐treatment technique that effectively reduces the interface recombination rate of charge carriers by adjusting the back contact of Sb_2_Se_3_ solar cells.^[^ ^54^ ^]^ In addition to TA‐based post‐treatment methods, many alternative approaches have been developed to modulate the optoelectronic properties of Sb_2_Se_3_, which are collectively referred to as TA‐free post‐treatment techniques in this review. This review highlights the breadth of strategies available for optimizing the performance of Sb_2_Se_3_ solar cells. Although modification operations regarding TA‐free post‐treatment techniques toward Sb_2_Se_3_ solar cells have been reported and successfully implemented, it is still challenging to fabricate reliable and high‐quality Sb_2_Se_3_ thin films and high‐efficiency devices under this condition.

### 2.3 Principal Functions of Post‐Treatment Techniques in the Fabrication of Sb2Se3 Layers

**Figure 2:**
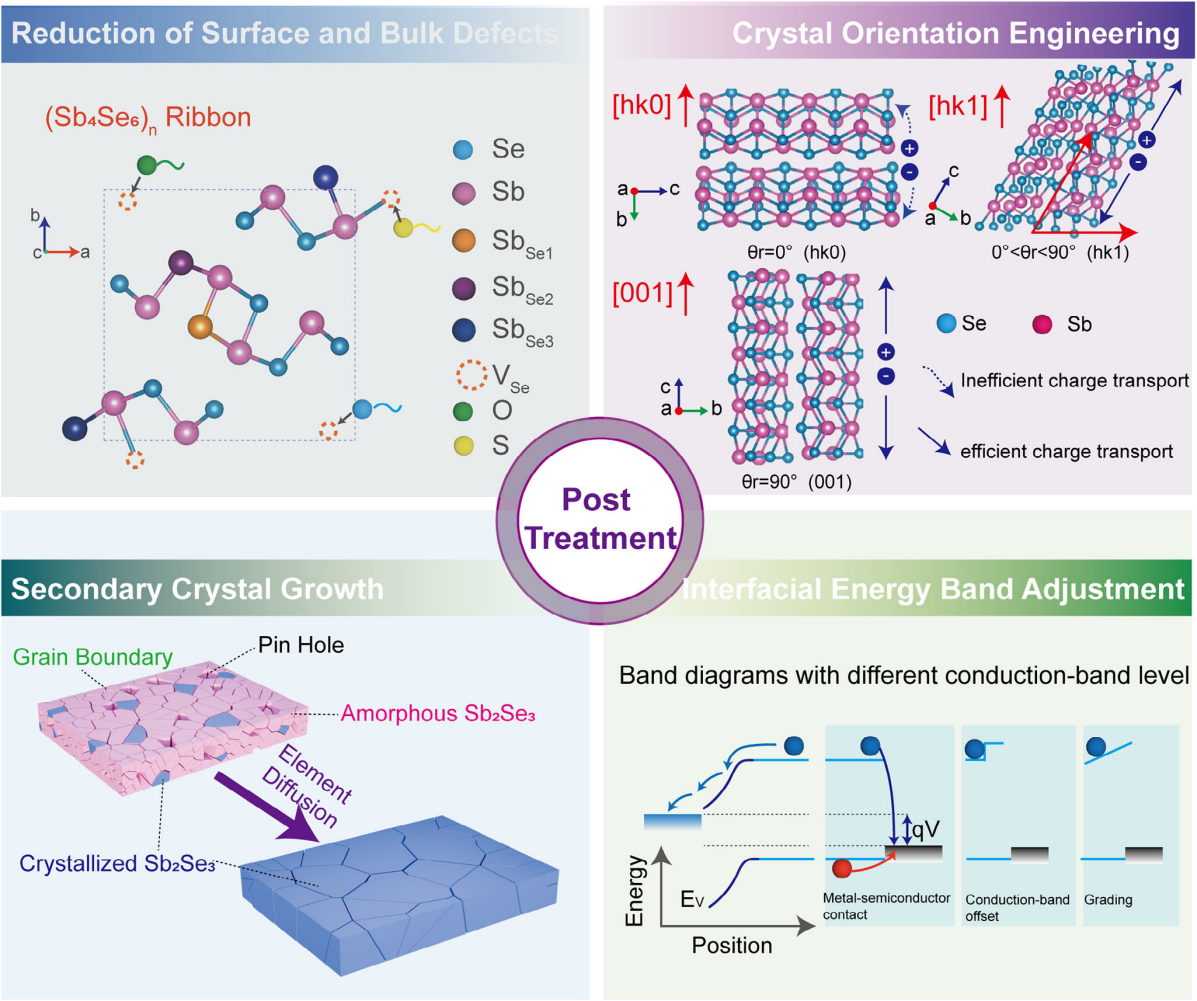
Principal functions of post‐treatment techniques in the fabrication of Sb_2_Se_3_ layers. a) A summary of the major defect passivation mechanisms. b) Schematic demonstration of [001]‐orientation growth of Sb_2_Se_3_ ribbons. c) Schematic demonstration of secondary grain growth process of Sb_2_Se_3_ grains. d) Schematic illustration of interfacial energy band adjustment.

With the continuous development of Sb_2_Se_3_ preparation technology, breakthroughs have been made in photovoltaic efficiency while improving the crystal quality of Sb_2_Se_3_ thin films. Specifically, various post‐treatment techniques are developed to enhance the film quality of Sb_2_Se_3_ photoactive layers from multiple perspectives. This section summarizes the principal functions of these post‐treatment techniques in the fabrication of Sb_2_Se_3_ layers, focusing on four key aspects: surface and bulk defect passivation, crystal orientation engineering, secondary crystal growth of Sb_2_Se_3_, and interfacial energy band adjustment (**Figure** **2**).

#### 2.3.1 Reduction of Surface and Bulk Defects

Sb_2_Se_3_ possesses an orthorhombic crystal structure which is composed of quasi‐1D [Sb_4_Se_6_]*_n_* ribbons arranged together via weak interactions.^[^ ^55^, ^56^, ^57^ ^]^ The low crystal symmetry of Sb_2_Se_3_ makes the chemical environment different for each Sb/Se element in the unit cell, resulting in two inequivalent Sb sites and three inequivalent Se sites. The unique quasi‐1D structure endows Sb_2_Se_3_ with complex deep‐level defect properties. Specifically, there are five types of vacancies (V_Sb(1)_, V_Sb(2)_, V_Se(1)_, V_Se(2)_, and V_Se(3)_ and five antisites (Se_Sb(1)_, Se_Sb(2)_, Sb_Se(1)_,Sb_Se(2)_, and Sb_Se(3)_). Besides, nine inequivalent sites for interstitials Sb_i_/Se_i_ are considered as initial defects.^[^ ^58^, ^59^ ^]^ The formation energies of defects change significantly as the growth conditions change from Sb‐rich to Se‐rich.^[^ ^60^, ^61^ ^]^ Under Sb‐rich conditions, Sb_Se_ and V_Se_ are the dominant defects due to their high concentrations (>10^14^ cm^−3^). Sb_Se_ shows particularly high concentration (at ≈10^15^ cm^−3^ level) because the formation energy of this defect is much lower than the formation enthalpy of Sb_2_Se_3_ phase. Evidently, these defects are ultradeep‐level defects with active energy much higher than 0.025 eV, which are hardly ionized but act as recombination centers, and ultimately lead to severe trap‐assisted SRH recombination and *V*_OC_ loss.^[^ ^62^ ^]^ While in Se‐rich films, these defects have relatively low concentrations and shallow energy levels, more promising for achieving highly efficient devices. Therefore, it is universally recognized that the passivation of structural defects by filling vacancies or fixing interstitial atoms by passivating agents is quite important in constructing high‐quality Sb_2_Se_3_ films. In addition, the irreversible oxidation processes starting from surface defects and grain boundaries often lead to poor interface contact and carries transport. It is worth noting that employing sulfur or selenium atmosphere for postannealing treatment can prevent the surface contamination, thereby effectively passivating surface even bulk defects of Sb_2_Se_3_ thin films. Moreover, post‐treatment operations can reduce structural defects by inhibiting nucleation and promoting crystal growth, while obtaining large‐sized grains. By healing detrimental Sb_2_Se_3_ defects, the nonradiative recombination at the Sb_2_Se_3_/carrier‐transport layer interface throughout the bulk of Sb_2_Se_3_ films can be reduced considerably, leading to significant enhancement of open‐circuit voltage (*V*_OC_).^[^ ^63^ ^]^

#### 2.3.2 Crystal Orientation Engineering

Sb_2_Se_3_ consists of 1D (Sb_4_Se_6_)*_n_* ribbons, which are stacked along the [001] direction through strong covalent Sb*─*Se bonds, but held together in the [100] and [010] directions by van der Waals forces.^[^ ^64^ ^]^ This structure leads to highly anisotropic electronic properties in Sb_2_Se_3_ crystals, which demands vertically aligned ribbons on a substrate to enable efficient charge transport,^[^ ^65^ ^]^ and thus achieve high‐efficiency Sb_2_Se_3_ solar cells. Furthermore, in the orthogonal direction, the parallel‐stacked ribbons produce almost no dangling bonds, even at grain boundaries (GBs). This feature effectively reduces recombination losses, which is a key advantage in photovoltaic applications.^[^ ^17^, ^19^, ^66^ ^]^ Therefore, orientation control is crucial for achieving efficient carrier transport and benign grain boundaries in Sb_2_Se_3_ films.

Crystal orientation engineering focuses on regulating nucleation and growth behavior to direct the growth of thin films along specific crystal planes. According to the principle of energy minimization, the (Sb_4_Se_6_)*_n_* ribbons tend to grow parallel to the substrate to minimize surface energy, which poses a significant challenge to promoting the growth of [hk1]‐oriented (Sb_4_Se_6_)*_n_* ribbons.^[^ ^67^, ^68^ ^]^ The postannealing temperature is one of the key factors determining the orientation of Sb_2_Se_3_ films. Specifically, introducing a slow‐heating annealing process before high‐temperature annealing can induce [hk1] orientation.^[^ ^69^ ^]^ This is due to the low‐temperature treatment stabilizing the morphology and orientation of the film, forcing the grains to maintain this state and grow uniformly during the subsequent high‐temperature annealing process.^[^ ^70^ ^]^ In addition, the annealing atmosphere is also one of the factors that dominate the crystal orientation of Sb_2_Se_3_. For instance, Sb_2_Se_3_ films annealed in H_2_S atmosphere predominantly expose the (020) plane, while films annealed in H_2_Se atmosphere tend to expose the (120) plane. In contrast, annealing in an inert Ar atmosphere is more conducive to exposing the (211) and (221) planes.^[^ ^48^ ^]^

#### 2.3.3 Secondary Crystal Growth of Sb2Se3

For polycrystalline thin‐film solar cells, passivation of GBs to suppress strong carrier recombination is mandatory to achieve high‐efficiency devices. Although the [001]‐oriented grains in Sb_2_Se_3_ thin films are associated with benign grain boundaries, the inevitable [hk0]‐oriented grains formed during the growth process of polycrystalline films can introduce detrimental grain boundaries which perform negative impact on device efficiency. Therefore, minimizing the GB density in Sb_2_Se_3_ thin films to obtain large and compact grains is necessary to reduce nonradiative recombination losses.^[^ ^71^ ^]^ In addition to deposition technologies, post‐treatment is the most widely used method for passivating grain boundaries, achieved by controlling grain size. For instance, creating a specific vapor environment to treat Sb_2_Se_3_ thin films can assist in grain growth, where the vapor used, such as sulfur, selenium, and oxygen, is typically sulfur homologues.^[^ ^72^ ^]^ These vapors create a favorable atmosphere for Sb_2_Se_3_ grains to recrystallize from the edges to supplement voids, increasing the grain size form hundreds of nanometers to micrometers. However, it should be noted that although vapor post‐treatments can effectively promote grain growth and GB passivation, they may also bring potential side effects. Excessive chalcogen vapor pressure or prolonged exposure time could lead to compositional nonuniformity, secondary phase formation, or interfacial instability due to elemental diffusion into adjacent layers. Therefore, careful optimization of parameters, such as vapor concentration, temperature, and treatment duration is crucial to balance grain enlargement with compositional integrity and interface quality.

#### 2.3.4 Interfacial Energy Band Adjustment

During the preparation of Sb_2_Se_3_, various defects with low formation energy are inevitably generated, which typically serve as charge traps facilitating trap‐assisted recombination.^[^ ^73^, ^74^ ^]^ Additionally, the capture of charges on the surface can create localized energy level offsets, resulting in undesirable energy band alignments and a reduction in built‐in potential (V_bi_).^[^ ^74^, ^75^ ^]^ Such mismatched energy levels arrangement leads to significant interfacial recombination, hindering carrier transport, and extraction at the interface, thereby limiting the photovoltaic performance of the device.

Nowadays, effective post‐treatment strategies toward Sb_2_Se_3_ films can not only passivate surface defects and assist in secondary grain growth, but also play an important role in adjusting the interfacial band structure, thereby achieving efficient carrier transport. Careful screening of post‐treatment reagents that match the Sb_2_Se_3_ crystal structure is an important means of effectively regulating chemical and energy level structure, both surface and bulk.^[^ ^60^, ^76^ ^]^ For example, Sb_2_Se_3_ films annealed in a sulfur atmosphere has been demonstrated to significantly increase the energy band.^[^ ^76^, ^77^ ^]^ Moreover, promoting the spatial separation of electron–hole pairs is an effective way to reduce the capture of charge carriers by deep‐level defect centers, which can be achieved by constructing an electric field between grain boundaries (GBs) and grain interiors (GIs).^[^ ^22^ ^]^ the distance between Sb_2_Se_3_ chains is large enough to accommodate most single atom cations or anions, doping is used to convert GB and GI into different carrier types, establishing a Fermi level difference between GB and GI, pushing electrons and holes toward GB and GI, respectively. Considering the distance between Sb_2_Se_3_ chains is sufficient (≈3.29 Å) to accommodate most single atom cations or anions, doping can be used to invert GB and GI into different carrier types to establish a Fermi level difference between them, pushing electrons and holes toward GBs and GIs, respectively.

In addition to post‐treatment and doping strategies, introducing a compositional bandgap gradient within the Sb_2_Se_3_ absorber layer has also been suggested as a promising route to further promote carrier separation and optimize interfacial energy level alignment, similar to approaches successfully implemented in CIGS and CdTe thin‐film solar cells. Although precise control of the bandgap gradient in Sb_2_Se_3_ remains challenging due to its unique quasi‐1D structure, combining mild compositional grading with appropriate post‐treatment methods may offer synergistic advantages for suppressing recombination and improving overall device efficiency (**Table** **1**).

**Table 1:** Photovoltaic parameters of recently reported Sb_2_Se_3_ solar cells fabricated by different post‐treatment techniques.

Post‐treatment techniques	*V*oc [V]	*J*sc [mA cm^−2^]	FF [%]	PCE [%]	Refs.
TA+O_2_ treatment	0.419	27.02	51.70	5.87	[70]
TA+H_2_S treatment	0.38	27.90	56.40	5.98	[49]
TA+LiOH treatment	0.41	30.5	60.51	7.57	[79]
TA+Sulfur treatment	0.47	29.10	59.41	8.22	[80]
TA+Sulfur treatment	0.448	30.36	65.44	8.90	[81]
TA+Sulfur treatment	0.430	28.22	61.71	7.49	[82]
TA+postselenization	0.408	26.3	31.32	3.36	[83]
TA+postselenization	0.42	19.30	45.12	3.57	[44]
TA+postselenization	0.335	24.4	46.8	3.7	[84]
TA+postselenization	0.57	10.2	55.3	3.22	[85]
TA+postselenization	0.45	31.65	57.93	8.25	[86]
Air annealing	0.517	24.80	59.44	7.62	[87]
TA+B_2_O_3_ treatment	0.461	30.57	66.52	9.37	[24]
TA+hydrogen‐assisted selenization	0.43	27.57	58.6	6.16	[88]
TA+CuCl_2_ treatment	0.413	28.1	54.4	6.32	[22]
TA+SbCl_3_ treatment	0.416	26.66	60.82	6.74	[89]
TA+KOH treatment	0.407	30.68	57.4	7.16	[90]
TA+(NH_4_)_2_S treatment	0.442	25.1	46.4	4.89	[91]
TA+CS_2_ treatment	0.40	29.2	47.5	5.55	[91]
TA+KCN treatment	0.382	21.7	63.4	5.3	[92]
TA+NH_3_ treatment	0.412	26.9	56.1	6.2	[93]
TA+HCl treatment	0.37	28.22	51.9	5.35	[94]
TA+P_2_O_5_ treatment	0.469	30.33	66.72	9.50	[25]
TA+Pt treatment	0.503	27.52	54.56	7.49	[95]
Low‐temperature preannealing +TA	0.407	12.11	30	1.47	[96]
TA in vacuum condition	0.371	26.01	55.7	5.72	[97]
Two‐step annealing	0.329	18.16	44.13	2.64	[98]
HTJ annealing treatment	0.52	27.8	59.8	8.64	[99]
HTJ annealing treatment+Copper acetate treatment	0.457	27.7	66.2	8.40	[100]
Photo Annealing	0.478	31.67	69.90	10.58	[101]
Liquid‐medium Annealing	0.470	30.74	64.37	9.28	[102]

## 3 Recent Progress of Post‐Treatment Techniques in Photovoltaic Application

As previously discussed, postprocessing techniques can be divided into two categories: TA‐based and TA‐free post‐treatment techniques. The development of TA‐based technology has systematically studied the effects of various adjustment factors, such as annealing atmosphere, temperature, and configuration, while retaining the core TA process. As for the TA‐free post‐treatment technology, it mainly uses substituted physical annealing and modulated chemical reactions to drive the crystallization behavior of Sb_2_Se_3_.

In this section, we comprehensively summarized the effects of diverse post‐treatment techniques on both the quality of Sb_2_Se_3_ films and the photovoltaic performance of devices. In addition, we analyzed the potential mechanisms of these treatments on the growth process of Sb_2_Se_3_, providing detailed insights into the structural, chemical, and electronic modifications.

### 3.1 TA‐Based Post‐Treatment Techniques

State‐of‐the‐art Sb_2_Se_3_ solar cells typically require thermal annealing (TA) at relatively high temperatures, ranging from 370 to 400 °C, to enhance the crystallinity and grain size of Sb_2_Se_3_ films. This process usually also involves adjusting the defect characteristics. Achieving uniformity and high crystallinity of thin films during the TA process is expected to fabricate highly efficient Sb_2_Se_3_ solar cells. In order to further optimize film quality, additional regulatory factors, such as incorporating a controlled chalcogen vapor atmosphere during TA and introducing chemical additives, are usually integrated with conventional TA treatments to fine‐tune the crystallization kinetics of Sb_2_Se_3_, thus improving overall device performance.

#### 3.1.1 Classical Thermal Annealing

Thermal annealing (TA) is commonly used in laboratories to promote the crystallization of Sb_2_Se_3_. Compared to traditional chalcogenide absorber materials, such as Cu(In,Ga)Se_2_ (melting point: 1300 K),^[^ ^103^ ^]^ CdTe (melting point: 1367 K),^[^ ^104^ ^]^ and Cu_2_ZnSnS_4_ (melting point: 1120 K),^[^ ^105^ ^]^ Sb_2_Se_3_ has a significantly lower melting point (885 K).^[^ ^106^ ^]^ This characteristic implies that the annealing temperature must be carefully controlled during the fabrication of Sb_2_Se_3_ thin film to avoid its evaporation or thermal decomposition. Excessive annealing temperature can lead to undesirable phenomena, such as forming intermediate species (e.g., Se_*n*_), which in turn introduce point defects and other structural imperfections into the film.^[^ ^107^ ^]^ Therefore, maintaining an appropriate annealing window is essential for minimizing the types and concentrations of deep‐level defects in Sb_2_Se_3_ films.

**Figure 3:**
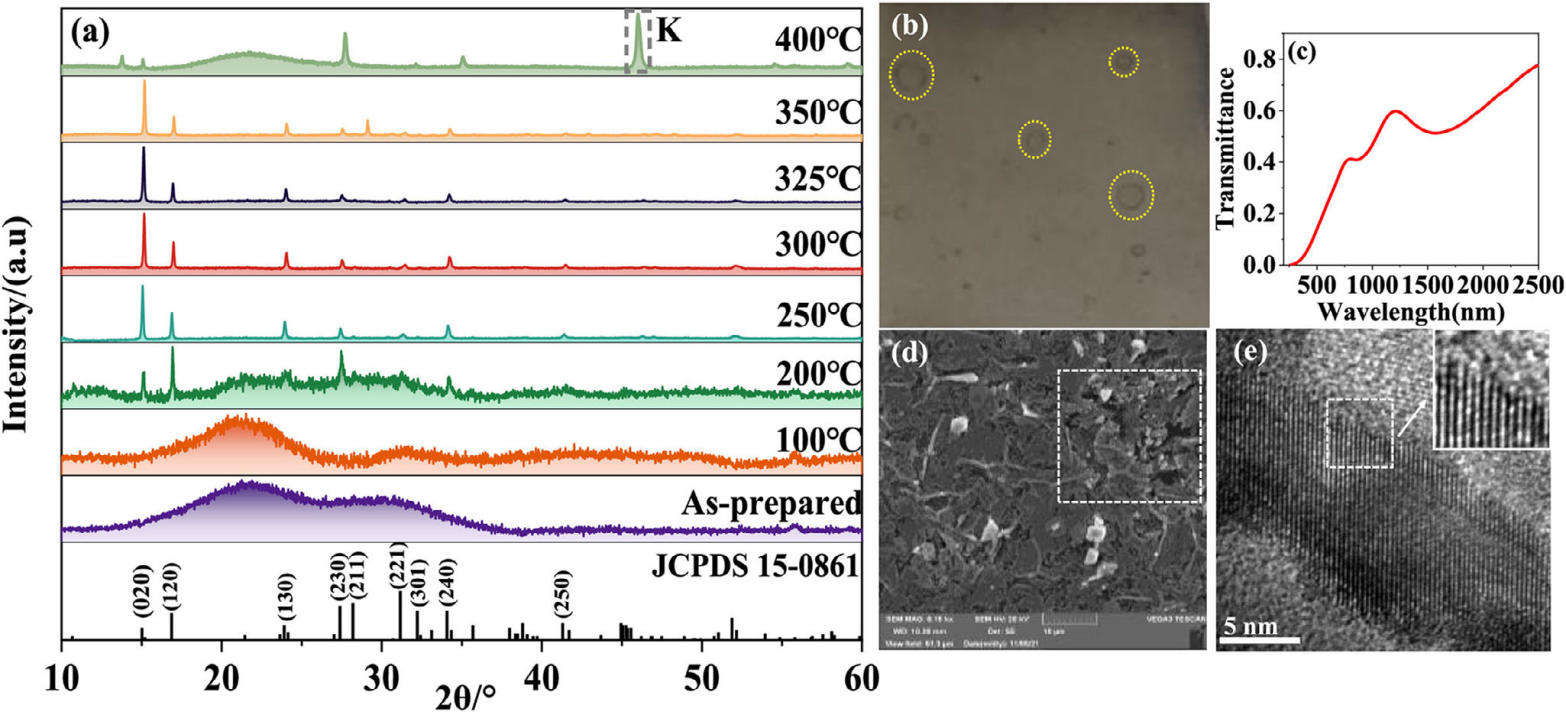
Surface morphology and structural characteristics of thin films. a) XRD diffraction patterns. b) Film surface after annealing at 400 °C. c) FTIR spectra of film surface after annealing at 400 °C. d) SEM image of thin film after annealing at 400 °C. e) HRTEM diagram of thin film after annealing at 400 °C. Reproduced with permission.^[^ ^41^ ^]^ Copyright 2024, Elsevier.

The initial main focus of postannealing is to promote the transformation of amorphous or poorly crystallized Sb_2_Se_3_ precursor films to well crystallized films, with the goal of achieving large grain sizes and vertical orientation.^[^ ^40^, ^57^ ^]^ Research has demonstrated that controlling the annealing temperature and duration is essential to achieving the optimal balance between crystallinity and compositional uniformity. Song et al. examined the thermally induced structural transformations of Sb_2_Se_3_ and demonstrated that low temperatures (about 100 °C) are not sufficient to fully crystallize the film (**Figure** **3**a).^[^ ^41^ ^]^ When the annealing temperature is higher than 200 °C, it can drive the crystallization of Sb_2_Se_3_ polycrystalline thin films, which is reflected as the appearance of X‐ray diffraction (XRD) peaks corresponding to specific crystallographic planes, including (020), (120), (130), (211), (221), (240), (250), and (061). This result emphasizes the strong dependence of the structural evolution of Sb_2_Se_3_ on the annealing temperature. However, additional structural and morphological changes occur when the annealing temperature exceeds 400 °C. For example, upon annealing at 400 °C, a dominant diffraction peak at 2θ = 46° (K position) related to Sb metal appears, suggesting the presence of significant selenium vacancies in the film. From the appearance, the film changes from silver gray to light yellow, forming bubbles on the surface (Figure 3b), indicating the gradual decomposition of the material. The surface defects displayed in SEM images (Figure 3d) and the blurred lattice fringes displayed in high‐resolution transmission electron microscopy (HRTEM) images (Figure 3e) further confirm the adverse effects of increasing annealing temperatures on the integrity of Sb_2_Se_3_ thin films. These results highlight the critical importance of optimizing annealing temperature in suppressing defects and ensuring film quality.

According to reports, the optimal crystallization temperature for Sb_2_Se_3_ is about 350 °C, which may vary by 10–20 °C depending on the preparation method. Deviations from this optimal range, whether by increasing the temperature or prolonging the annealing duration, leads to the decomposition of Sb_2_Se_3_, causing the film to deviate from stoichiometry and resulting in overall performance deterioration. Therefore, controlling the annealing temperature and duration is crucial for producing Sb_2_Se_3_ thin films with desirable structure and optoelectronic properties.

#### 3.1.2 Reactive Annealing

**Figure 4:**
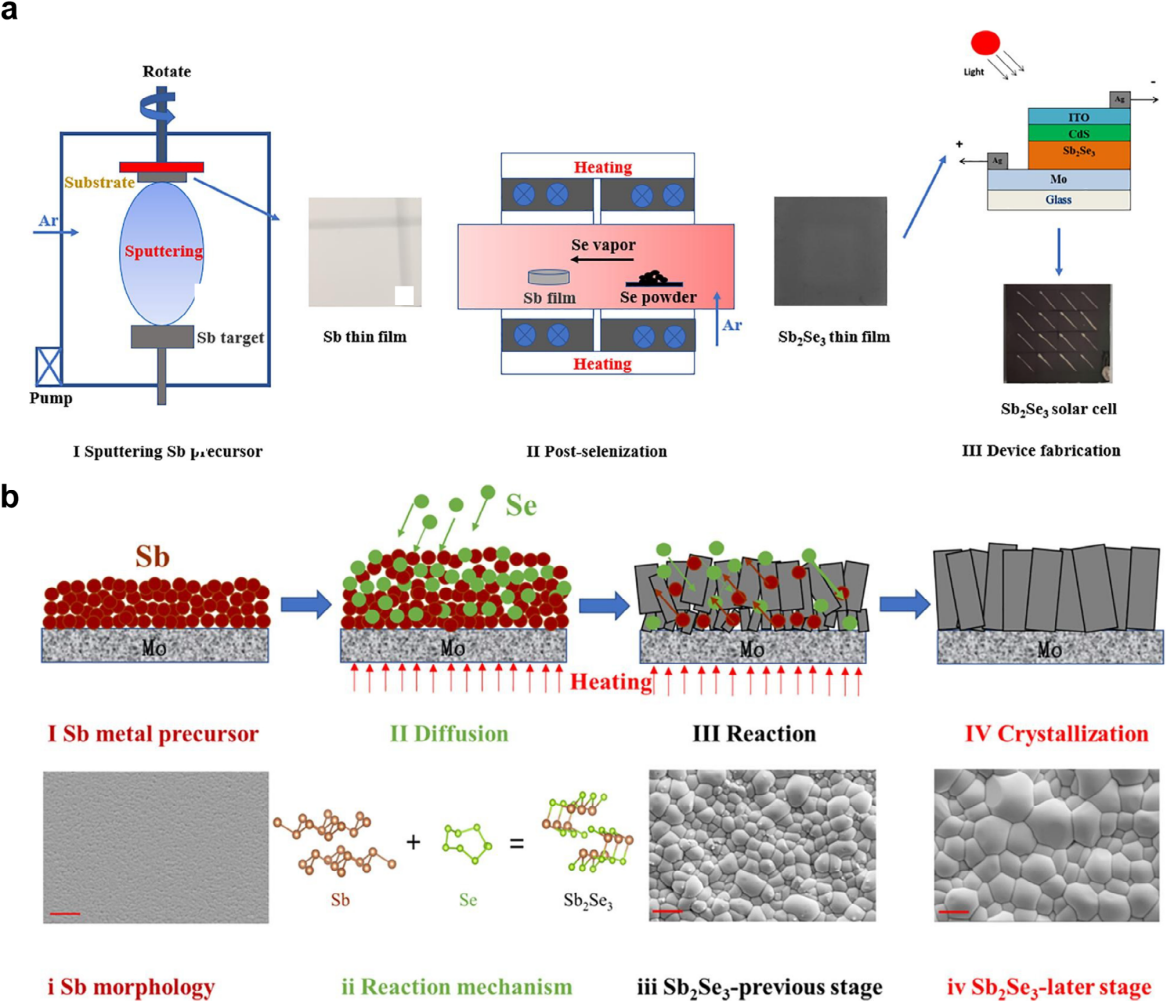
a) Schematic diagram of the Sb_2_Se_3_ device fabrication involves sputtering Sb precursor and postselenization. b) Crystallization procedures of Sb_2_Se_3_ thin films prepared via Sb metallic precursor selenization. Reproduced with permission.^[^ ^111^ ^]^ Copyright 2020, Elsevier.

Sputtering followed by postannealing is widely employed in the fabrication of copper indium gallium selenide (CIGS),^[^ ^108^ ^]^ copper zinc tin sulfide (CZTS),^[^ ^109^ ^]^ and copper zinc tin sulfur selenide (CZTSSe)^[^ ^110^ ^]^ thin‐film solar cells. This method has also been applied in the preparation of Sb_2_Se_3_, which involves high‐energy annealing reaction. The specific steps are to anneal the presputtered Sb metal precursor film under Se vapor (**Figure** **4**a).^[^ ^111^ ^]^ During the annealing process, Sb films are exposed to Se atmosphere at elevated temperatures, facilitating the formation of Sb_2_Se_3_ through diffusion and reaction. This reaction annealing process offers the advantage of precise control over both the morphology and composition of the films. By optimizing the annealing process, this method achieves an improvement in the crystallinity of the absorber layer and an increase in grain size, successfully preparing self‐assembled Sb_2_Se_3_ thin films consisting of large grains spanning the entire film thickness. Additionally, the selenization process enhances film quality and device performance in two key ways: 1) compensating for selenium loss during deposition, which reduces selenium vacancy (V_Se_)‐related recombination;^[^ ^112^ ^]^ and 2) forming a thin MoSe_2_ layer at the Sb_2_Se_3_/Mo interface in the substrate structure based on Mo glass, eliminating Schottky barrier and reducing recombination at the back interface.^[^ ^36^ ^]^

The substrate temperature plays a crucial role in achieving the optimal morphology, crystallinity, and crystal orientation of Sb_2_Se_3_ thin films. Liang et al. studied the detailed structural evolution by adjusting the temperature of substrate carrying Sb metal layer from 340 to 460 °C.^[^ ^113^ ^]^ It is worth noting that the thin film annealed at temperatures below 400 °C has poor crystallization with small Sb_2_Se_3_ grains distributing on the surface. At 460 °C, the obtained film consists of rod‐shaped Sb_2_Se_3_ grains oriented parallel to the substrate and embedded with some microvoids. In contrast, an annealing temperature of 420 °C achieved optimal crystallinity, chemical composition, and crystal orientation. Finally, Sb_2_Se_3_ grains with stoichiometry, vertical orientation, and size exceeding 1 µm were obtained by radio frequency (RF) magnetron sputtering. This is because the selenization temperature deviating from the optimal value can lead to insufficient crystallization or decomposition of Sb_2_Se_3_ films, which are not suitable for device applications. Liang et al. further investigated the effect of selenization duration^[^ ^111^ ^]^ and revealed that the phase formation process includes four stages: preparation of Sb metal precursor (stage Ι), element diffusion (stage II), reaction (stage III), and crystallization (stage IV) (Figure 4b). Specifically, the first 5–15 min of selenization are the stage of crystal growth, during which the grain size rapidly increases. Prolonged annealing does not significantly affect crystallinity as the grain growth stops. It should be noted that insufficient or excessive annealing time can cause the film to deviate from stoichiometric composition, resulting in Se‐poor or Se‐rich states, respectively. A selenization duration of 15 min is beneficial for achieving an optimal Sb/Se ratio of 0.67. Therefore, the temperature and duration of selenization are closely associated with the quality of Sb_2_Se_3_ crystals. As a result, the method of predepositing Sb metal layers followed by selenization post‐treatment achieves [001] orientation, which increases the open circuit voltage of the solar cell to 500 Mv.^[^ ^114^ ^]^

#### 3.1.3 Thermal Annealing Combined with Defects Engineering

Thermal annealing (TA) treatment is widely employed to facilitate the crystallization of Sb_2_Se_3_, but this process often leads to a series of defects in the film from microscopic to macroscopic levels. The microscopic defects primarily include point defects such as vacancies, interstitials, and antisites, while macroscopic defects encompass 2D grain boundaries (GBs), 3D cracks and pinholes, which can be observed through optical microscopy and scanning electron microscopy (SEM). Among the point defects, the most common species—V_Se_, V_Sb_, Se_Sb_, and Sb_Se_—generate trap states within the conduction band (CB) and valence band (VB) of Sb_2_Se_3_. These point defects are capable of migrating through macroscopic defects such as GBs. According to current research, the high‐density trap states in Sb_2_Se_3_ may be the primary cause of voltage losses in Sb_2_Se_3_‐based solar cells.^[^ ^115^ ^]^

Defects in the absorber layer diminish device performance through trap‐assisted carrier recombination, specifically via Shockley–Read–Hall (SRH) recombination. Although TA treatment is effective for promoting crystallization, it is insufficient to address the microscopic defects within the material. As such, defect engineering, in conjunction with TA treatment, has become a standard approach for producing high‐crystallinity, low‐defect‐density Sb_2_Se_3_ films, which plays a vital role in the currently reported high‐efficiency Sb_2_Se_3_ solar cells. This effect can be divided into two categories: physical process and chemical process. The physical process involves postprocessing and introduction of effective passivators into the bulk of Sb_2_Se_3_ under thermal drive. The chemical process is mainly achieved by depositing reactive chemical reagents on Sb_2_Se_3_, which can not only react chemically with defects but also cover the entire Sb_2_Se_3_ layer. In order to passivate deep‐level defects, various passivators, including inorganic elements and their corresponding salts, are utilized in the post‐treatment process. These passivation strategies alleviate nonradiative recombination in the device, thereby enhancing the open‐circuit voltage (*V*_oc_) and improving the short‐circuit current (*J*_sc)_ and fill factor (FF) by enhancing interface contact.^[^ ^63^, ^95^ ^]^

**Figure 5:**
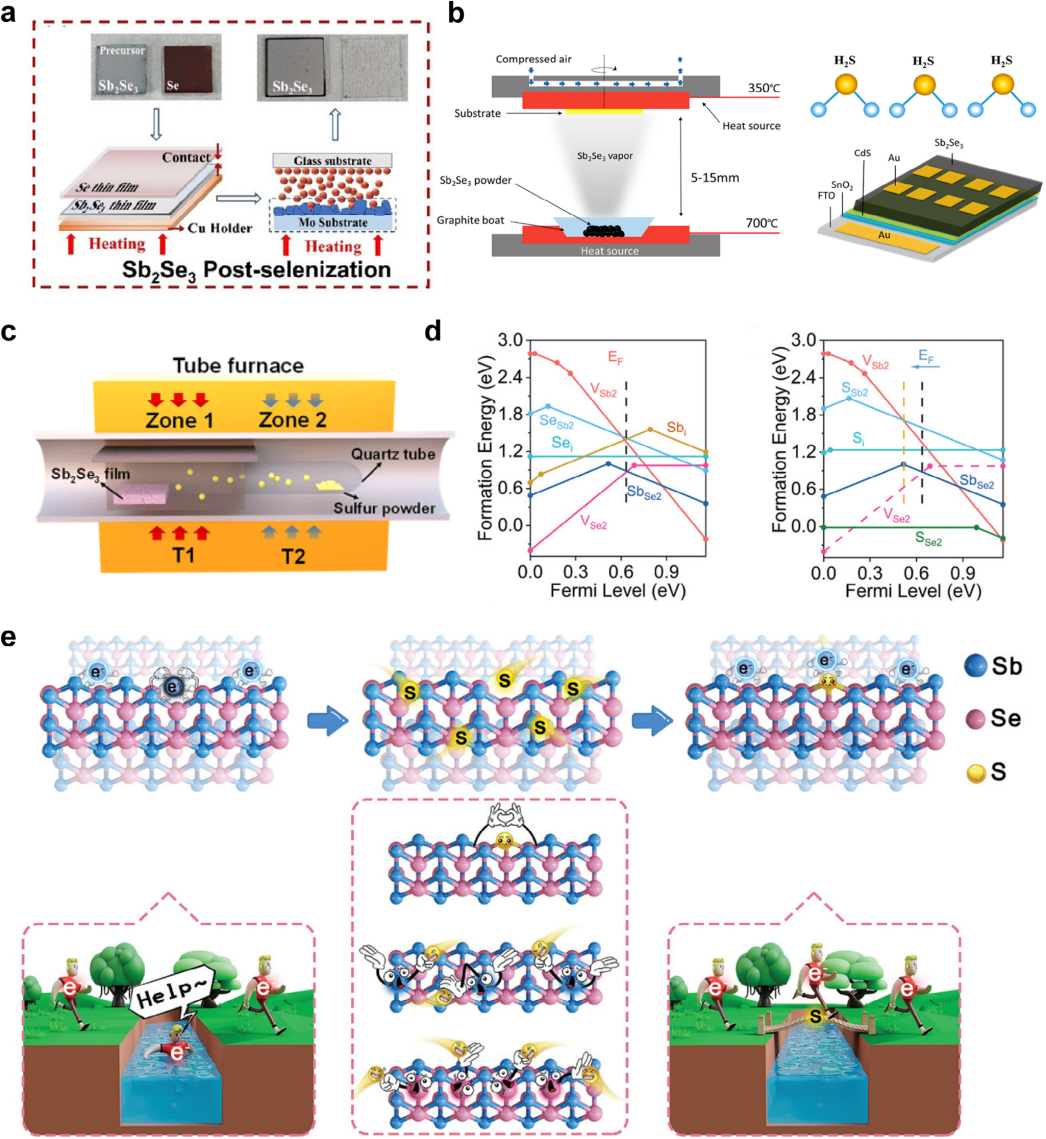
a) Postselenization and regrowth of Sb_2_Se_3_ thin film. Reproduced with permission.^[^ ^44^ ^]^ Copyright 2024, Elsevier. b) Preparation of the Sb_2_Se_3_ films using H_2_S. Reproduced with permission.^[^ ^49^ ^]^ Copyright 2020, American Chemical Society. c) The as‐designed postsulfurization apparatus for the fabrication of Sb_2_Se_3_ films. Reproduced with permission.^[^ ^80^ ^]^ Copyright 2024, Royal Society of Chemistry. d) Calculated formation energy of intrinsic defects as a function of Fermi level in Sb_2_Se_3_ under the Sb‐rich condition and calculated formation energy of the typical intrinsic defects with low formation energy and sulfur‐doped defects as a function of Fermi level in Sb‐rich Sb_2_Se_3_. e) Schematic diagram of the mechanism of defect passivation. Reproduced with permission.^[^ ^81^ ^]^ Copyright 2024, Wiley.

As mentioned earlier, the predominant physical strategy for defect passivation involves introducing passivators into the bulk of Sb_2_Se_3_ films driven by heating. According to theoretical calculations, vacancies and antisites are the dominant point defects in Sb_2_Se_3_, with concentrations exceeding 10^12^ cm^−3^. However, V_Se_ is the only intrinsic point defect with high concentrations under both Sb‐rich and Se‐rich conditions, which is extremely harmful to the optoelectronic properties of the film. Therefore, suppressing vacancies, particularly V_Se_, is critical for improving device performance. Homologous elements with electronic structures similar to Se atoms have a passivating effect on V_Se._^[^ ^20^ ^]^ Introducing excess Se into the Sb_2_Se_3_ bulk phase to transform the film from an Sb‐rich to a Se‐rich state is the most direct method to passivate V_Se_. For Sb_2_Se_3_ solar cells with a substrate structure, post‐selenization has become a standard procedure to fabricate high‐crystalline, low‐concentration defect films. In the selenization process, Se powder is commonly used as the precursor material which typically exists in the relatively inactive form of Se_6_ rings.^[^ ^116^ ^]^ At conventional annealing temperatures of 300–400 °C, the postselenization process is difficult to completely eliminate harmful V_Se_ defects in Sb_2_Se_3_ films. Therefore, precise control of postselenization conditions, including adjusting environmental vacuum degree, selenization temperature, and selenization duration, is required. Recently, Liang et al. developed an innovative postselenization method,^[^ ^44^ ^]^ in which the predeposited Se layer on the surface of Sb_2_Se_3_ thin films is uniformly incorporated into Sb_2_Se_3_ through thermal diffusion (**Figure** **5**a). Raising the temperature helps with the diffusion of Se, thereby addressing the problem of Se deficiency, reducing defect density, and minimizing the probability of carrier capture.

Sulfurization also offers a promising strategy for passivating V_Se_ in Sb_2_Se_3_ due to the similar atomic structure of sulfur to selenium. The highly reactive hydrogen sulfide (H_2_S) has been shown to provide better sulfurization effects for fabricating Sb_2_Se_3_. Li et al. conducted postannealing treatment on Sb_2_Se_3_ films using H_2_S atmosphere, effectively removing the oxide layer from the film surface while compensating for element losses^[^ ^49^ ^]^ (Figure 5b). X‐ray photoelectron spectroscopy (XPS) results demonstrate that S atoms have been successfully introduced into the Sb_2_Se_3_ thin film. Theoretical calculations indicate that the incorporation of S atom increases the electronic interaction with Sb atom, leading to a reduction in vacancy defects. However, the high toxicity and corrosiveness of H_2_S increases the complexity of use and increases safety risks. As an alternative, low‐toxic sulfur powder is used as a sulfur source. Considering the low reactivity of S powder, precise control of annealing parameters (such as configuration, temperature, duration, and sulfur vapor pressure) is crucial to improve the passivation effect of sulfur powder. Recently, Chen et al. proposed two sulfurization schemes which successfully produced high‐quality Sb_2_Se_3_ films with smooth, compact surface and favorable growth orientations. The first approach focuses on optimizing the sulfurization process by modifying the sulfurization configuration (Figure 5c), which involves connecting a quartz tube to a graphite box. Due to the confined space, only a small amount of sulfur powder can ensure the uniform diffusion of sulfur vapor, while maintaining a high sulfur partial pressure. Compared to conventional TA processes, gas‐phase sulfur facilitates rapid element transfer during Sb_2_Se_3_ grain growth, resulting in larger grains and fewer grain boundary defects. This sulfurization treatment not only induces [hk1] preferred crystal orientation, but also significantly reduces defect types and decreases capture cross‐section, which helps to enhance photovoltaic performance. The second sulfurization scheme involves a lower‐temperature treatment using thermal evaporation at 280 °C for 5 min.^[^ ^81^ ^]^ Due to the introduction of only a small amount of sulfur (0.8 at% more than control Sb_2_Se_3_), the crystal structure, morphology, bandgap, and composition of Sb_2_Se_3_ films does not undergo significant changes. However, this sulfurization method successfully transforms detrimental V_Se_ defects into benign S_Se_ defects, thereby reducing defect‐assisted recombination and prolonging carrier lifetime (Figure 5d,e).

**Figure 6:**
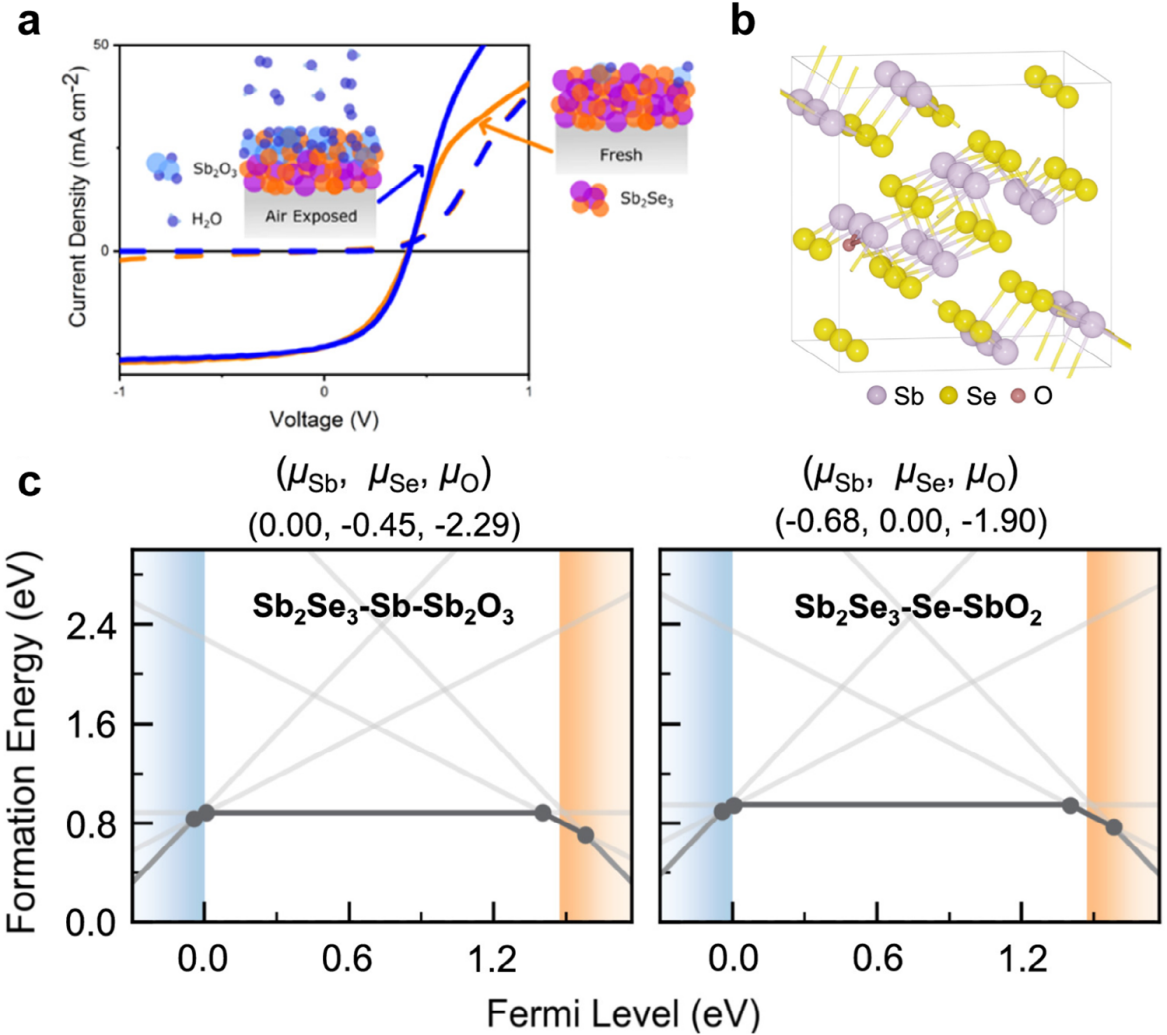
a)  Current–voltage plots of a freshly made reference cell and cells based on Sb_2_Se_3_ exposure to oxygen. Reproduced with permission.^[^ ^78^ ^]^ Copyright 2020, American Chemical Society. b–d) Calculated defect properties of oxygen substitution in Sb_2_Se_3_. Reproduced with permission.^[^ ^20^ ^]^ Copyright 2024, Elsevier.

Oxygen substitution presents a promising strategy for mitigating the impact of selenium (Se) vacancies in Sb_2_Se_3_. Major et al. demonstrated that exposing Sb_2_Se_3_ to oxygen can improve the performance of solar cells, and speculated from the atomic structure that it is caused by O atoms filling harmful Se vacancies.^[^ ^78^ ^]^ Walsh et al. studied the structural configuration and formation energy of O substituted Se sites (O_Se_) through theoretical calculations.^[^ ^20^ ^]^ They demonstrated that the neutral state of O_Se_ is thermodynamically stable throughout almost the entire bandgap, resulting in shallow defect energy levels that do not cause carrier recombination. Under oxygen‐poor conditions, the formation energy of O_Se_ is relatively low (≈0.8 eV), comparable to the formation energy of V_Se_. These results highlight the potential role of oxygen in passivating Se vacancies and mitigating their detrimental effects by converting deep‐level and recombination‐active V_Se_ into shallow and inactive states (**Figure** **6**). However, exposure to oxygen can easily lead to the formation of antimony oxide on the Sb_2_Se_3_ surface, which may hinder the interface contact between Sb_2_Se_3_ and the hole transport layer. Therefore, precise control of oxygen partial pressure is essential when using oxygen as a defect passivator.

Due to the lack of clear criteria for screening passivators, the current physical defects engineering for Sb_2_Se_3_ only considers atomic structure, resulting in insufficient selection of more effective passivators. Therefore, it is necessary to improve the molecular selection criteria for defect passivation of Sb_2_Se_3_ films in future research.

The most conventional chemical treatments for Sb_2_Se_3_ thin films usually involves a chemical reaction to passivate surface defects. The importance of this strategy has been well demonstrated in related photovoltaic technologies, such as Cu(In,Ga)Se_2_ and kesterite solar cells.^[^ ^117^ ^]^ Surface chemical treatment plays a crucial role in defect passivation and interface optimization. Among various chemical treatments, chemical etching has emerged as the predominant means for surface passivation and interface modification in Sb_2_Se_3_ solar cells. For example, studies have shown that post‐treatment of Sb_2_Se_3_ with ammonium sulfide ((NH_4_)_2_S) can passivate surface defects, thereby reducing recombination losses and improving the interface quality between the absorber layer and the hole transport layer. This is because the S atoms in (NH_4_)_2_S can react with Se vacancies or other defect states on the surface of Sb_2_Se_3_, effectively reducing defect density and improving the dynamic transport of charge carriers. Moreover, the surface chemistry state of the film is modified to achieve better band alignment, which is beneficial for facilitating efficient charge transfer at the heterojunction.^[^ ^24^, ^25^, ^118^ ^]^

As mentioned earlier, suppressing charge recombination caused by unfavorable grain boundaries (GBs) in polycrystalline thin films is another essential factor in optimizing the optoelectronic performance of Sb_2_Se_3_‐based devices.^[^ ^119^ ^]^ In Sb_2_Se_3_ polycrystalline films, interstitial and vacancy defects are prone to occur in the GBs and act as centers for nonradiative recombination, which hinder the carrier transport.^[^ ^67^, ^71^ ^]^ Chemical treatments such as depositing passivation layers or ion exchange to passivate GB have shown great potential in reducing recombination losses and enhancing device performance.

One effective strategy to address GB passivation involves the deposition of protective capping layers, such as spin coating inorganic salts. Recently, Tao et al. innovatively proposed an environmentally friendly and cost‐effective method of using antimony trichloride (SbCl_3_) as a passivator to treat Sb_2_Se_3_ thin films.^[^ ^89^ ^]^ Research has shown that SbCl_3_ solution can effectively fill the voids in GBs and produce a denser and more uniform morphology. This process reduces the film roughness, and improves the contact between the absorber layer and the electrode, thereby minimizing leakage current and suppressing recombination at the back interface. This improvement enhances the photoelectric response in the long‐wavelength region, leading to notable improvements in both the fill factor (FF) and short‐circuit current density (*J*_sc_).

**Figure 7:**
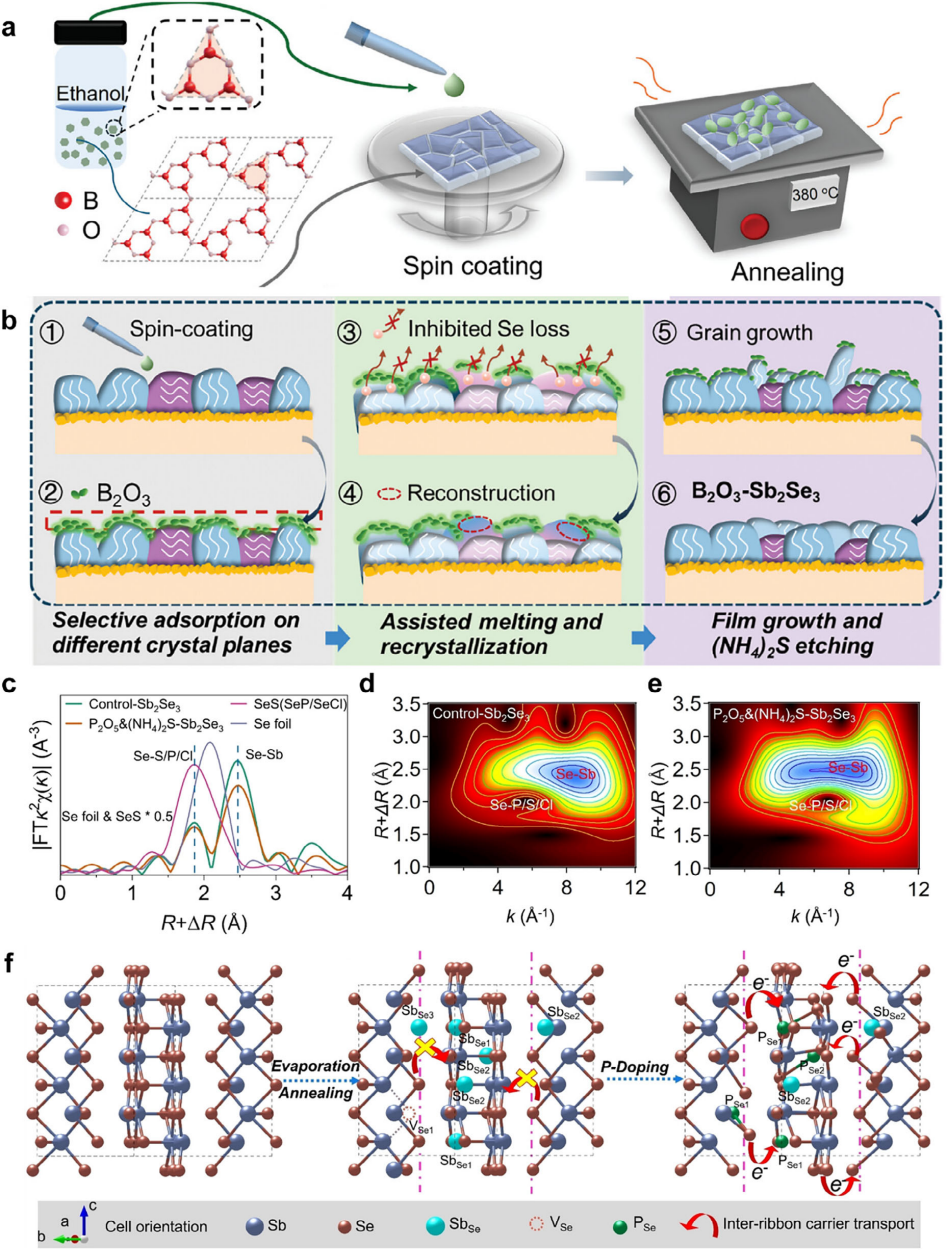
a) Spin coating of B_2_O_3_ solution on the surface of Sb_2_Se_3_ film, followed by annealing at 380 °C.  b) Schematic diagram of the crystallization process for B_2_O_3_‐Sb_2_Se_3_ film. Reproduced with permission.^[^ ^24^ ^]^ Copyright 2024, Wiley. c) The *k* ^2^‐weighted Fourier transformed Se *K*‐edge spectra for Se foil, SeS, Control‐Sb_2_Se_3_, and P_2_O_5_&(NH_4_)_2_S‐Sb_2_Se_3_ films. Wavelet transform contour spectra of Se *K*‐edge EXAFS data for d) Control‐Sb_2_Se_3_ and e) P_2_O_5_&(NH_4_)_2_S‐Sb_2_Se_3_ films. f) Schematic diagram of defects, dimensionality and carriers transport characteristics of Control‐Sb_2_Se_3_ and P_2_O_5_&(NH_4_)_2_S‐Sb_2_Se_3_. Reproduced with permission.^[^ ^25^ ^]^ Copyright 2024, Wiley.

Besides, our previous research demonstrated that interactions between interface materials and Sb_2_Se_3_ significantly influence the film's crystallization behavior. Building on this understanding, we developed a simple yet effective postdeposition treatment using boron oxide (B_2_O_3_) to regulate Sb_2_Se_3_ crystallization^[^ ^24^ ^]^ (**Figure** **7**a). The B_2_O_3_ layer coating on the film surface acts as a flux, promoting recrystallization at the interface. Mechanistic studies reveals that B_2_O_3_ preferentially adsorbed on the (hk1) planes of Sb_2_Se_3_, inducing vertical orientation growth. On the other hand, the B*─*Se bond formed between B_2_O_3_ and Sb_2_Se_3_ inhibits the volatilization of Se, which suppresses V_Se_ defects (Figure 7b). The synergistic effect of orientation control and defect passivation improves photogenerated carrier separation and transport. The resulting B_2_O_3_‐treated solar cells achieves a PCE of 9.37%, representing a 14.1% improvement over untreated devices.

Certain passivation agents can not only form protective surface layers but also diffuse into the absorber bulk to achieve doping effects. In contrast to B_2_O_3_ regulating interfacial crystallization, we employed phosphorus pentoxide (P_2_O_5_) as an alternative post‐treatment reagent, which enabled the diffusion of P elements into the bulk of Sb_2_Se_3_ absorber through heat treatment.^[^ ^25^ ^]^ Via this method, P doping is ultimately achieved in the thin film. Wavelet transform (WT) analysis of Se K‐edge EXAFS reveals distinct coordination signatures in P_2_O_5_&(NH_4_)_2_S‐treated Sb_2_Se_3_ films (Figure 7c–e). The WT contour plots display intensity peaks corresponding to Se‐Sb and Se‐P/S/Cl coordination. These results show that the P_2_O_5_ treatment slightly elongated the bond lengths of Se‐Sb and Se‐P/S/Cl in the film, and reduce the coordination number of Se‐Sb, indicating that the doping of P promotes lattice distortion and replaces partial Sb atoms to form bonds with Se atoms. Importantly, the P incorporation decreases the spacing between (Sb_4_Se_6_)*_n_* ribbons, thereby enabling multidirectional (3D‐like) carrier transport across the quasi‐1D ribbon structure (Figure 7f). Moreover, the P_Se_ antisite defects introduced by P doping have lower formation energy, effectively passivating intrinsic defects such as V_Se_ vacancies and Sb_Se_ antisites, and improving the overall dynamic transport of carriers in the material.

**Figure 8:**
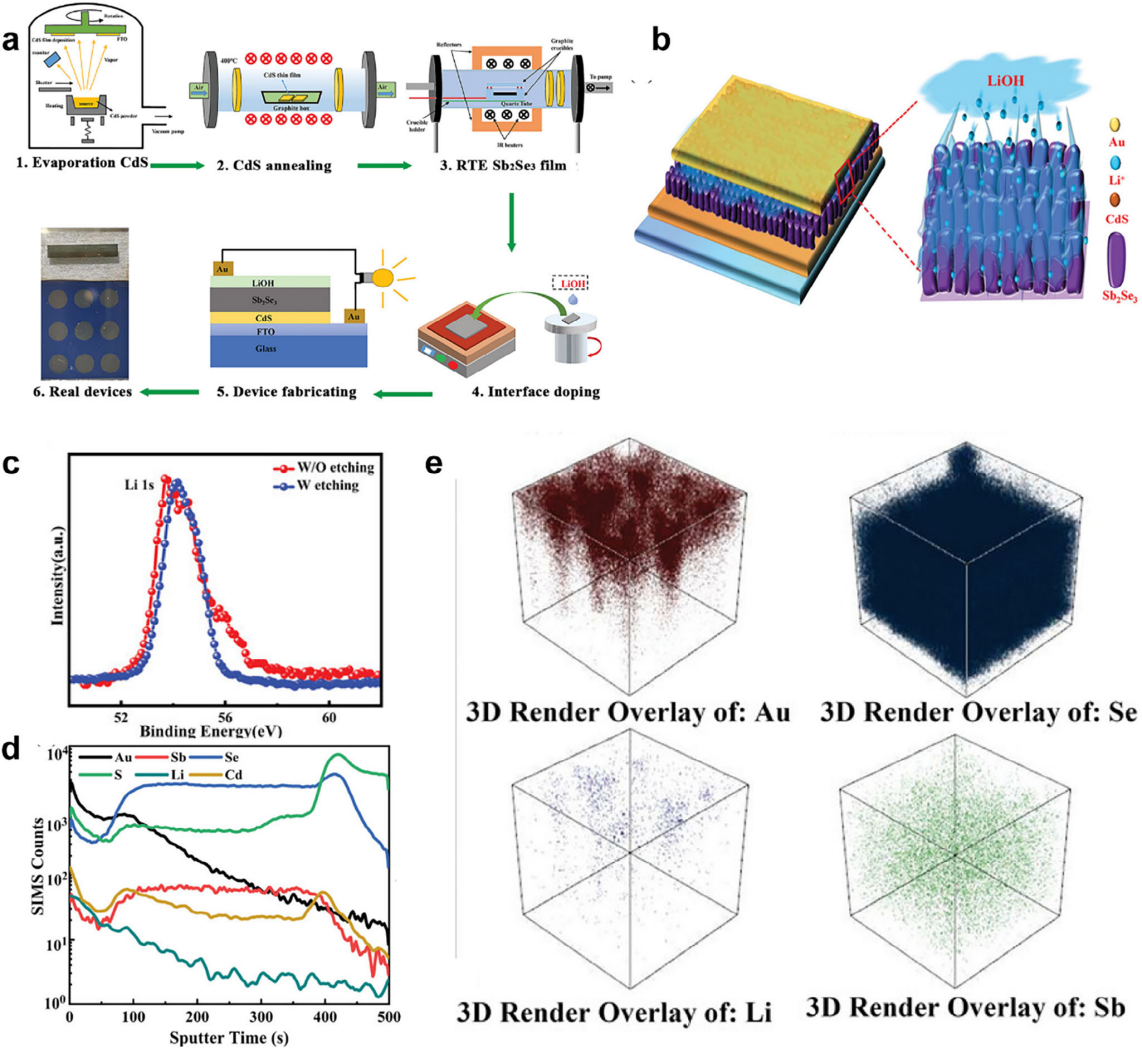
a) Illustration of the fabrication process for Sb_2_Se_3_ solar cells with Li gradient field by upper interface doping via solution method. b) The schematic diagram of a Li‐doped Sb_2_Se_3_ film. c) XPS patterns of the Sb_2_Se_3_ film with etching. d) TOF−SMIS patterns of the Sb_2_Se_3_ film with Li gradient field, e) 3D‐rendered overlays of selected elements of Sb_2_Se_3_ film with Li gradient field. Reproduced with permission.^[^ ^79^ ^]^ Copyright 2023, Wiley.

Ding et al. successfully passivated the surface defects of Sb_2_Se_3_ thin films using lithium hydroxide (LiOH)^[^ ^79^ ^]^ (**Figure** **8**a,b). Mechanistic study shows that low concentration of LiOH induces mild surface etching, removes surface defects, and allows Li⁺ ions to diffuse into the bulk film (Figure 8c). This treatment produces a smoother and more uniform surface with fewer pinholes, thereby reducing light‐induced recombination at the absorber/metal interface. Moreover, X‐ray photoelectron spectroscopy (XPS) and time‐of‐flight secondary ion mass spectrometry (TOF‐SIMS) confirm that Li⁺ can diffuse along the (Sb_4_Se_6_)*_n_* molecular chains and establish a lithium gradient field through upper‐interface doping (Figure 8d). The gradient distribution of Li causes the Fermi level of Sb_2_Se_3_ to shift toward the conduction band in a gradient form, forming a gradient conduction‐band‐minimum (CBM) structure which effectively reduces the potential barrier and facilitates the transport of charge carriers across GBs. In addition, the introduction of Li leads to a reduction in defect density and GB inversion, ultimately suppressing carrier recombination and enhancing carrier collection.

Similarly, Ding et al. demonstrated that treatment with potassium hydroxide (KOH) solution not only etches the surface of Sb_2_Se_3_ thin films, but also increases the doping density through diffusion of K ions in the films.^[^ ^90^ ^]^ Meanwhile, KOH treatment improves the back contact properties of the solar cells, which is attributed to the surface etching of thin Sb_2_O_3_ layer by KOH solution.

**Figure 9:**
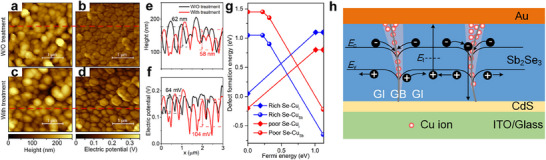
KPFM measurement on Sb_2_Se_3_ with and without CuCl_2_ treatment. a,c) Surface morphology of Sb_2_Se_3_ without and with CuCl_2_ treatment, respectively. b,d) Surface potential of Sb_2_Se_3_ without and with CuCl_2_ treatment, respectively. e,f) Corresponding height and electric potential profiling plots along the red dashed lines in (a–d). g) Calculated formation energies of the Cu dopants on different doping sites, Cu_Sb_ and Cu_i_, under Se‐rich (blue) and Se‐poor (red) conditions. Cu_i_ is an n‐type shallow defect with a 0.11 eV depth below the conduction band. h) Schematic diagram of carrier kinetics and band bending in a typical Sb_2_Se_3_ grain with CuCl_2_ treatment. Reproduced with permission.^[^ ^22^ ^]^ Copyright 2018, American Chemical Society.

Many inorganic salts have been proven to act as passivators. For instance, Tang et al. reported a significant improvement in the efficiency of Sb_2_Se_3_ solar cells by inverting GB properties through Cu doping.^[^ ^22^ ^]^ In this approach, Sb_2_Se_3_ films were treated with an ammonia (NH_3_·H_2_O) solution of CuCl_2_, which controlled the release rate of Cu^2^⁺ ions by forming copper ammonia complexes ([Cu(NH_3_)_4_]^2^⁺). Due to the lower compactness of GBs compared to grain interiors (GIs), Cu^2^⁺ ions mainly diffuse along GBs, thereby achieving uniform and mild passivation. Kelvin probe force microscopy (KPFM) characterization and density functional theory (DFT) calculations confirm that Cu doping shifted the Fermi level Sb_2_Se_3_ upward by introducing interstitial Cu (Cu_i_) defects, and convert GBs to n‐type conductivity (**Figure** **9**a–g). The spatial separation of photogenerated carriers suppress recombination losses and significantly enhance device performance (Figure 9h).

#### 3.1.4 Heterojunction Annealing

Heterojunction annealing plays a pivotal role in enhancing the performance of substrate‐structured Sb_2_Se_3_ solar cells by improving the crystallinity and lattice matching between the absorber and buffer layers (such as CdS).^[^ ^120^, ^121^, ^122^ ^]^ This process reduces defect states, especially interface defects, by regulating atomic rearrangement and element interdiffusion at the interface, optimizes band alignment, and achieves effective charge separation and transfer. Furthermore, annealing the heterojunction stabilizes the heterojunction interface, ensuring the long‐term stability of the device while improving power conversion efficiency. By addressing key limitations, such as interfacial recombination and defect density, HTJ annealing technique is indispensable for achieving higher power conversion efficiencies and advancing the development of next‐generation solar cell technologies.

**Figure 10:**
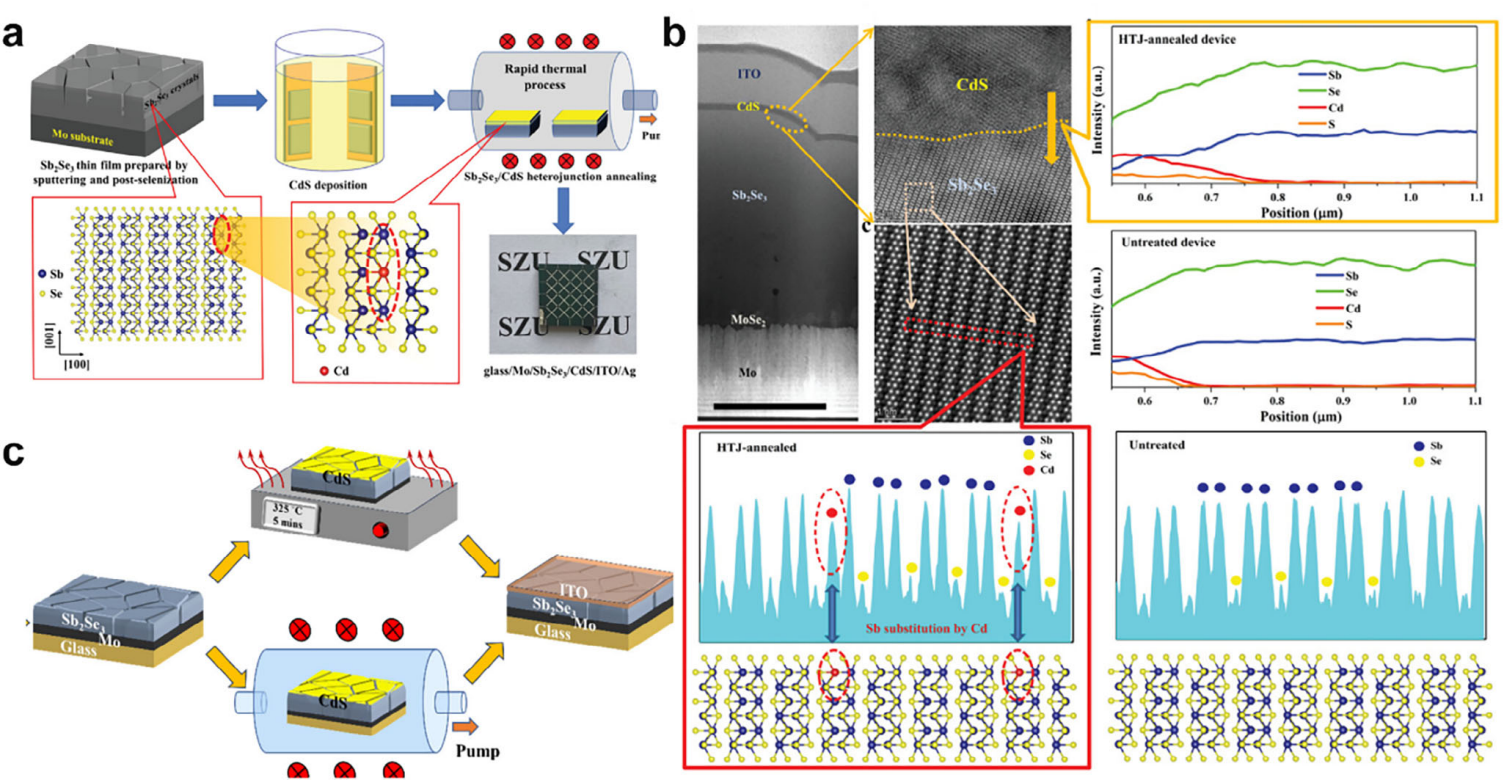
a) Schematic diagram of the rapid thermal processing under vacuum. b) TEM characterization of the HTJ‐annealed device. Reproduced with permission.^[^ ^99^ ^]^ Copyright 2023, Wiley. c) Schematic diagram of the rapid thermal processing under vacuum and air annealing. Reproduced with permission.^[^ ^87^ ^]^ Copyright 2024, MDPI.

In this field, Tang et al. reported an effective interface engineering approach for postannealing Sb_2_Se_3_/CdS heterojunction (HTJ) using vacuum rapid thermal processing (RTP)^[^ ^99^ ^]^ (**Figure** **10**a). This annealing method induces Cd diffusion from CdS buffer layer to Sb_2_Se_3_ absorber, resulting in Cd substitution for Sb in the near‐surface region. This means that the treatment successfully introduces shallow Cd_Sb_ hole traps, increases doping concentration, and thus expands the built‐in potential (V_bi_) of the device (Figure 10b). Transmission electron microscopy (TEM) shows that the indistinct Sb_2_Se_3_/CdS interface observed after annealing indicated a reduction in lattice mismatch and fewer interface defect states. Additionally, deep defects within the space charge region (SCR) of Sb_2_Se_3_ are effectively passivated, suppressing nonradiative recombination at the HTJ and extending the minority carrier lifetime. These improvements ultimately result in a power conversion efficiency (PCE) of 8.64% and a record *V*_OC_ of 0.52 V for Sb_2_Se_3_/CdS thin‐film solar cell with substrate structures.

Tang et al. also investigated the effect of air atmosphere on heterojunction annealing^[^ ^87^ ^]^ (Figure 10c). The capacitance–voltage (C–V) and drive‐level capacitance profiling (DLCP) characterizations confirm that the defects at the Sb_2_Se_3_/CdS interface are effectively passivated, improving the interface transport properties of the device. This result further demonstrates the effectiveness of the annealing process in improving device performance.

### 3.2 TA‐Free Post‐Treatment Techniques

The energy source of traditional thermal annealing (TA) comes from heat, which is a simple and convenient method for producing Sb_2_Se_3_ films of various sizes. However, the key issue with this method is the incompatibility between flexible substrates (such as polyethylene terephthalate (PET)) and high‐temperature annealing (over 100 °C).^[^ ^123^ ^]^ This constraint significantly hinders the integration of Sb_2_Se_3_ films into flexible and lightweight solar cells. Therefore, developing alternative TA‐free post‐treatment technique which is capable of producing high‐quality Sb_2_Se_3_ films is expected to further promote the commercialization of Sb_2_Se_3_ solar cells.

At present, there are extremely limited postprocessing strategies for TAs. It is worth mentioning that this method has played a comparable role to the TA method in controlling the crystallization behavior and internal microstructure of thin films.

**Figure 11:**
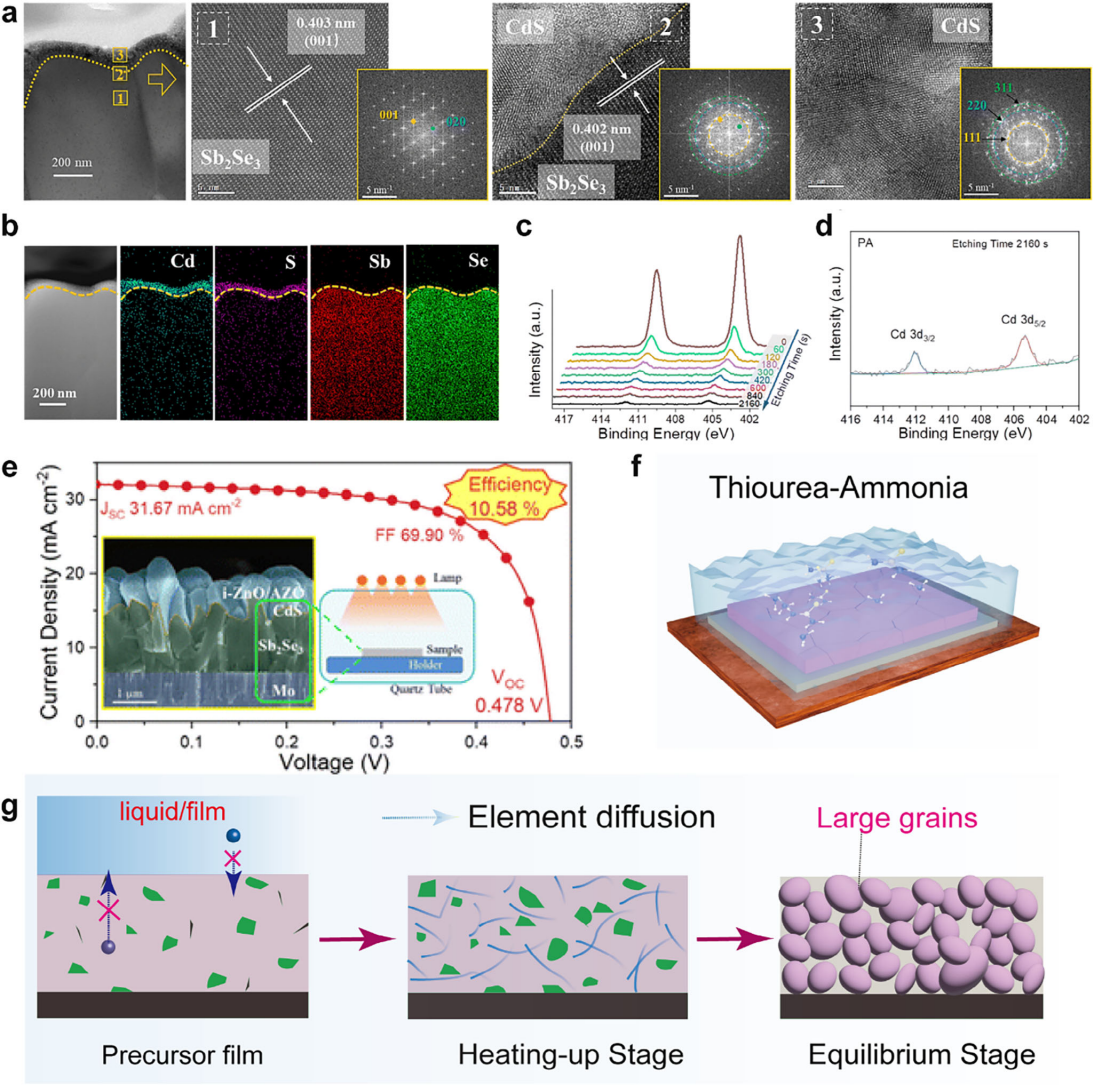
Characterization of the CdS/Sb_2_Se_3_ junction: a) cross‐sectional images and the corresponding selected‐area high resolution TEM images. b) energy‐dispersive spectroscopy elemental mapping; and c) in‐depth Cd 3d XPS spectra of the photoannealing sample with different etching times. d) The Cd 3d spectra of the photoannealing sample with an etching time of 2160 s. e) *J–V* curves of photoannealing Sb_2_Se_3_‐based solar cells. Reproduced with permission.^[^ ^101^ ^]^ Copyright 2024, The Royal Society of Chemistry. f,g) Diagram of the liquid medium annealing (LMA) process. Reproduced with permission.^[^ ^102^ ^]^ Copyright 2023, Wiley.

For example, Li et al. developed an innovative photoannealing approach to enhance the Sb_2_Se_3_/CdS heterojunction interface.^[^ ^101^ ^]^ In this method, Sb_2_Se_3_ absorbers were deposited onto Mo‐coated glass substrates via an injection vapor deposition (IVD) technique, followed by the deposition of CdS electron transport layer. Under a pressure of 50 kPa in a vacuum chamber, the Sb_2_Se_3_/CdS heterojunction was subjected to photoannealing for 30 min. This process facilitates the diffusion of Cd from the CdS layer into the Sb_2_Se_3_ absorber. Transmission electron microscopy (TEM) (**Figure** **11**a,b) and X‐ray photoelectron spectroscopy (XPS) (Figure 11c,d) results confirm the successful Cd diffusion, which promotes atomic rearrangement and improved lattice matching at the heterojunction interface. The incorporation of Cd into Sb_2_Se_3_ bulk reduces deep‐level defects and forms a high‐quality absorber layer. Furthermore, drive‐level capacitance profiling (DLCP) and deep‐level transient spectroscopy (DLTS) measurements demonstrate significant suppression of nonradiative recombination at both the interface and within the bulk absorber, particularly mitigating Sb_Se_‐related hole traps. These improvements enhance the transport and collection of photogenerated carriers, resulting in increased *V*_OC_, *J*_SC_, and FF. As a result, the optimized Sb_2_Se_3_/CdS heterojunction achieves a PCE of 10.58%, with a certified efficiency of 10.18%, which is the highest efficiency reported for Sb_2_Se_3_ solar cells to date.

More recently, our group developed a novel annealing approach that utilized an ammonia‐thiourea mixed solution as a liquid medium to regulate the crystallization of Sb_2_Se_3_ films at a lower temperature of 66 °C^[^ ^102^ ^]^ (Figure 11f,g). This liquid‐medium annealing (LMA) method enabled the preparation of Sb_2_Se_3_ films with large grain size and enhanced [hk1] orientation. By analyzing the morphology and microstructure evolution of the thin film, potential grain growth mechanisms were elucidated. In this method, the growth of grains is mainly attributed to the rapid heat transfer characteristics of the liquid medium, which promotes the unique crystallization kinetics of the thin film. In addition, the alkaline environment of the solution promotes grain activation and the release of S^2−^ ions from thiourea decomposition, which in turn cause grain fusion to form micrometer sized grains with preferential orientations. Moreover, the liquid medium provides a uniform growth environment for the film, effectively mitigating anion loss and reducing deep‐level defects. Therefore, the LMA technique has resulted in a champion PCE of 9.28% for Sb_2_Se_3_ solar cells, demonstrating the potential of LMA as a scalable low‐temperature method in fabricating high‐performance devices.

In conclusion, although there is limited research on the TA‐free strategy, existing studies have demonstrated the enormous potential of this method in achieving controlled crystallization and improving film quality, which can rival traditional TA related techniques. However, further exploration and development of innovative technologies are urgently needed for both methods to achieve the fabrication of high‐performance Sb_2_Se_3_ solar cells, thereby realizing their flexible and economically efficient applications.

## 4 Summary and Perspectives

This review provides a systematic summary of recent advancements in post‐treatment techniques for Sb_2_Se_3_ films and their applications in enhancing photovoltaic device efficiency. A brief review is conducted on the established vapor deposition methods, including one‐step and two‐step sequential deposition, to emphasize the critical role of post‐treatment processes throughout the Sb_2_Se_3_ fabrication process. The latest progress in post‐treatment approaches, encompassing both TA‐related and TA‐free strategies, is highlighted, with a focus on their critical contributions to the fabrication of high‐quality Sb_2_Se_3_ thin films. These contributions include passivation of surface and bulk defects, as well as regulation of crystal orientation, secondary grain growth, and interfacial energy band alignment. Given the dominant position of TA‐related techniques, this review comprehensively analyzes their technological development and applications in photovoltaics, while also exploring the emerging potential of TA‐free methodologies.

Recent advances in manufacturing processes have propelled the efficiency of Sb_2_Se_3_ solar cells beyond 10%, with innovative post‐treatment strategies playing a decisive role in achieving high‐quality and precise design of Sb_2_Se_3_ films. The postprocessing techniques afford fine control over the crystallization dynamics of Sb_2_Se_3_, enabling tailored optimization of film properties. Methods such as TA treatments and liquid medium annealing are increasingly being employed in combination to synergistically regulate the crystallization process. The preparation of smooth, defect‐free, [hk1]‐oriented, and highly crystalline Sb_2_Se_3_ films remains one of the key goals for achieving their commercialization. For this purpose, future research should address the following key challenges and directions.

A deeper understanding of Sb_2_Se_3_ nucleation and crystal growth under specific postprocessing conditions is imperative. Although substantial progress has been made in elucidating the formation mechanism of Sb_2_Se_3_ phase during TA treatment, further research is required to decode the intricate relationship between processing parameters (such as temperature, annealing atmosphere, gas pressure, and chemical additives) and crystallization kinetics. These factors have a crucial impact on the mesoscopic morphology of Sb_2_Se_3_ films, which directly governs device performance. Employing state‐of‐the‐art in situ characterization techniques is essential for unraveling these processes and guiding the and guiding the preparation of highly crystalline, pinhole‐free Sb_2_Se_3_ thin films.

TA‐based post‐treatment techniques have always maintained a dominant position in the fabrication of high‐quality Sb_2_Se_3_ thin films and high‐efficiency photovoltaic devices. However, their scalability is often challenged by temperature uniformity, thermal source stability, and environmental sensitivity of certain procedures (including sulfurization and selenization processes), which pose obstacles to commercial applications. Therefore, developing facile, reliable, and reproducible TA‐based techniques that can address the scalability issues while maintaining high device performance is crucial for practical applications.

Conversely, research on TA‐free post‐treatment methods is in its infancy, resulting in photovoltaic efficiency lagging behind that attained through TA‐based approaches. It is worth noting that the TA‐free technique shows considerable potential in finely controlling the crystallization kinetics of Sb_2_Se_3_. Given the respective advantages of both methods, it is crucial to develop a synergistic postprocessing strategy that could reconcile the trade‐offs between film quality, processing complexity, and industrial viability.

In addition to Sb_2_Se_3_ absorber layers, functional layers such as electron transport layers (ETLs) and hole transport layers (HTLs) also require tailored postprocessing to optimize charge transport properties. For example, TA treatment is indispensable for fabricating high‐quality CdS or TiO_2_‐based ETLs. However, these steps increase the complexity of device fabrication. Developing post‐treatment strategies that can simultaneously optimize the optical and electrical properties of Sb_2_Se_3_ layer and carrier transport layer is able to simplify device manufacturing. More importantly, exploring novel fabrication routes to produce high‐quality Sb_2_Se_3_ films without any post‐treatment could revolutionize the manufacturing paradigm.

In conclusion, advanced post‐treatment techniques are crucial for enhancing the multifunctional properties of Sb_2_Se_3_ thin films and achieving superior photovoltaic performance. Despite significant progress in TA‐based and TA‐free strategies, challenges remain in balancing film quality, process efficiency, and industrial feasibility. Addressing these challenges is key to driving the industrial application of Sb_2_Se_3_‐based optoelectronic devices. Continued development of post‐treatment technology is expected to accelerate progress in this area, ultimately enabling the commercialization of next‐generation Sb_2_Se_3_ solar cells (Supporting Information).

## Conflict of Interest

The authors declare no conflict of interest.

## Supporting information



Supporting Information

## Data Availability

The data that support the findings of this study are available from the corresponding author upon reasonable request.
